# Adult Jejunojejunal Intussusception Caused by Small Bowel Leiomyoma

**DOI:** 10.7759/cureus.63587

**Published:** 2024-07-01

**Authors:** Samuel G Romanini, Jose C Ardengh

**Affiliations:** 1 Gastrointestinal Endoscopy, Hospital Nove de Julho, São Paulo, BRA; 2 Gastrointestinal Endoscopy, Hospital das Clínicas de Ribeirão Preto, Ribeirão Preto, BRA; 3 Image Diagnosis, Universidade Federal de Sao Paulo, São Paulo, BRA

**Keywords:** jejunojejunal intussusception, treatment choices, accurate diagnosis, intestinal invagination, leiomyoma, intestine obstruction, intestine intussusception, surgery, acute abdomen

## Abstract

This article reports a case of a patient with intestinal obstruction admitted to the hospital whose presumed diagnosis by CT was jejunojejunal intussusception. The patient underwent exploratory laparotomy with an enterectomy of the invaginated segment. The histopathological and immunohistochemical results of the surgical specimen confirmed the presence of a small bowel leiomyoma. This case highlights the importance of a detailed clinical evaluation of patients with an intestinal obstruction who seek emergency care. The cause of intestinal obstruction is a diagnostic challenge due to the numerous pathologies that can lead to the development of the condition. Guided anamnesis, detailed physical examinations, and accurate subsidiary exams that do not delay diagnosis are the cornerstones of emergency room care. Knowing the ideal time to refer the patient to the operating room requires knowledge and practice. The patient reported in this article with jejunal leiomyoma as a cause of intestinal intussusception is surprising for its rarity and illustrates the range of pathologies that can lead to intestinal obstruction.

## Introduction

Intussusception is the insertion of part of the intestine into its adjacent distal portion. The invaginated intestine retracts the mesentery with its blood vessels, which leads first to venous and then arterial stasis. Intussusception is more frequent in childhood, and the highest incidence is recorded between the second and 12th months of life [1]. In adults, this phenomenon is a rare clinical entity, commonly diagnosed during surgeries performed for intestinal obstruction. The division of intussusception into juvenile and adult has great repercussions regarding the etiology, presentation of the clinical condition, time to diagnosis, and therapeutic approach [1].

Juvenile invaginations are mostly idiopathic, that is, there is no organic lesion that works as a lead point; they are functional disorders [1]. Exceptionally, intussusception occurs as a result of developmental abnormalities such as Meckel diverticulum, Henoch-Schonlein purpura, or lymphoma [1]. The clinical condition is most often characteristic, and the suspicion of intussusception can be established by clinical examination. To confirm the diagnosis, the method of choice is ultrasonography, which achieves excellent results in terms of speed and diagnostic accuracy. Regarding the therapeutic approach, there is a general consensus that non-operative reduction of intussusception (under fluoroscopy or ultrasound control) is recommended when there are no absolute contraindications, which include suspected intestinal perforation, peritonitis, hypovolemic shock, and onset of discomfort for more than 24 hours [1].

Invaginations in adults are in more than 95% of cases caused by organic lesions, most often tumors [1]. Malignant tumors predominate in intussusception. The clinical picture is not specific; thus, the triad of characteristic symptoms, which includes abdominal pain, enterorrhagia, and a palpable mass, is not present. There are also subclinical variants, as well as intermittent non-obstructive intussusception. If we keep in mind that the occurrence of intussusception in adults is extremely rare and often not taken into account as a hypothesis, it is clear why the diagnosis is often made intraoperatively.

However, the available diagnostic procedures, mainly ultrasound and CT scans, significantly improved the percentage of preoperative diagnoses of intussusception. As much as there is a diagnostic dilemma when a lot of evidence suggests that there is intussusception in an adult, this is an indication of surgical intervention [1]. The division of the intussusception can also be done by location, that is, according to the height of the intussusception, as this mechanism is described throughout the small intestine, including the duodenum and colon.

Certainly, intussusception occurs more frequently in the ileum, such as ileojejunal and ileoileal; the colon-ileal (ceco-ileal) and jejunojejunal forms are rare [1]. The authors report a case of jejunojejunal intussusception caused by a primary leiomyoma of the jejunal wall, which was successfully treated surgically on an emergency basis.

## Case presentation

**Figure 1:**
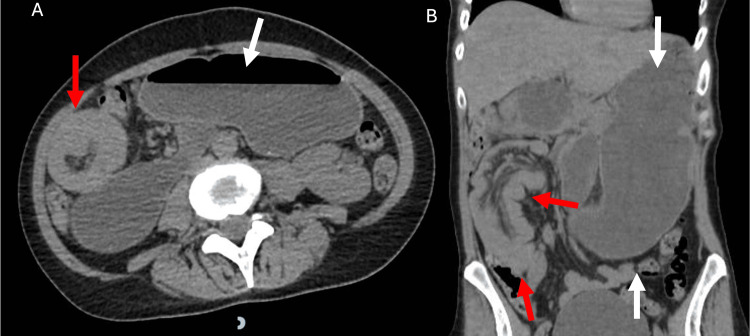
(A) Axial abdominal CT showing the target signal (red arrow) with significant liquid distension of the stomach (white arrow). (B) Coronal view of the abdominal CT with a jejunojejunal intussusception on the right side of the abdomen (red arrow) and the enormous distension of the stomach and duodenum (white arrow) CT: computed tomography

A 52-year-old female patient with a history of bipolar disorder sought emergency medical care due to a five-day history of diffuse, recurrent abdominal pain with progressive worsening, associated with nausea, vomiting, and constipation. She had no fever and no history of previous surgeries. Abdominal physical examination revealed distension, auscultation without air-fluid noises, severe pain on palpation, and rectal examination without signs of bleeding or fecal residue. Laboratory tests showed leukocytosis (17,600/mm³), increased creatinine (1.5 mg/dL), increased C-reactive protein (7 mg/dL), and hyponatremia (128 meq/L). She underwent an abdominal CT, which showed jejunojejunal intussusception of about 13.5 cm on the right side of the abdomen, associated with significant liquid distension of the duodenum and stomach (Figure 1).

**Figure 2:**
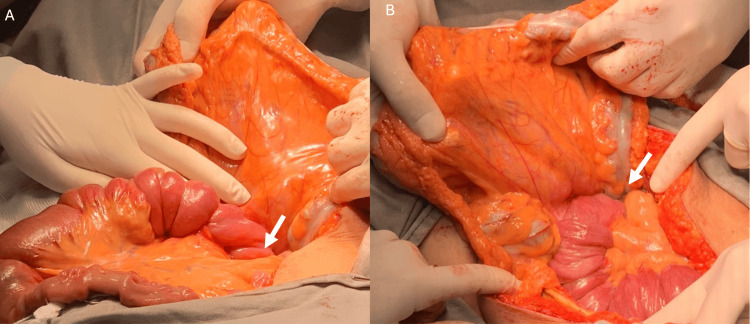
Intraoperative view of the (A) jejunojejunal intussusception (white arrow). (B) After the maneuver, the presence of a tumor in the jejunal wall was noted (white arrow)

As a supportive treatment, intravenous hydration, antibiotic therapy, analgesia, and antiemetics were performed, in addition to the passage of a nasogastric tube with an immediate output of 1800 ml of enteric content. An emergency exploratory laparotomy was indicated due to an obstructive acute abdomen. Intraoperatively, intussusception of jejunal loops at 20 cm from the angle of Treitz was observed (Figure 2).

**Figure 3:**
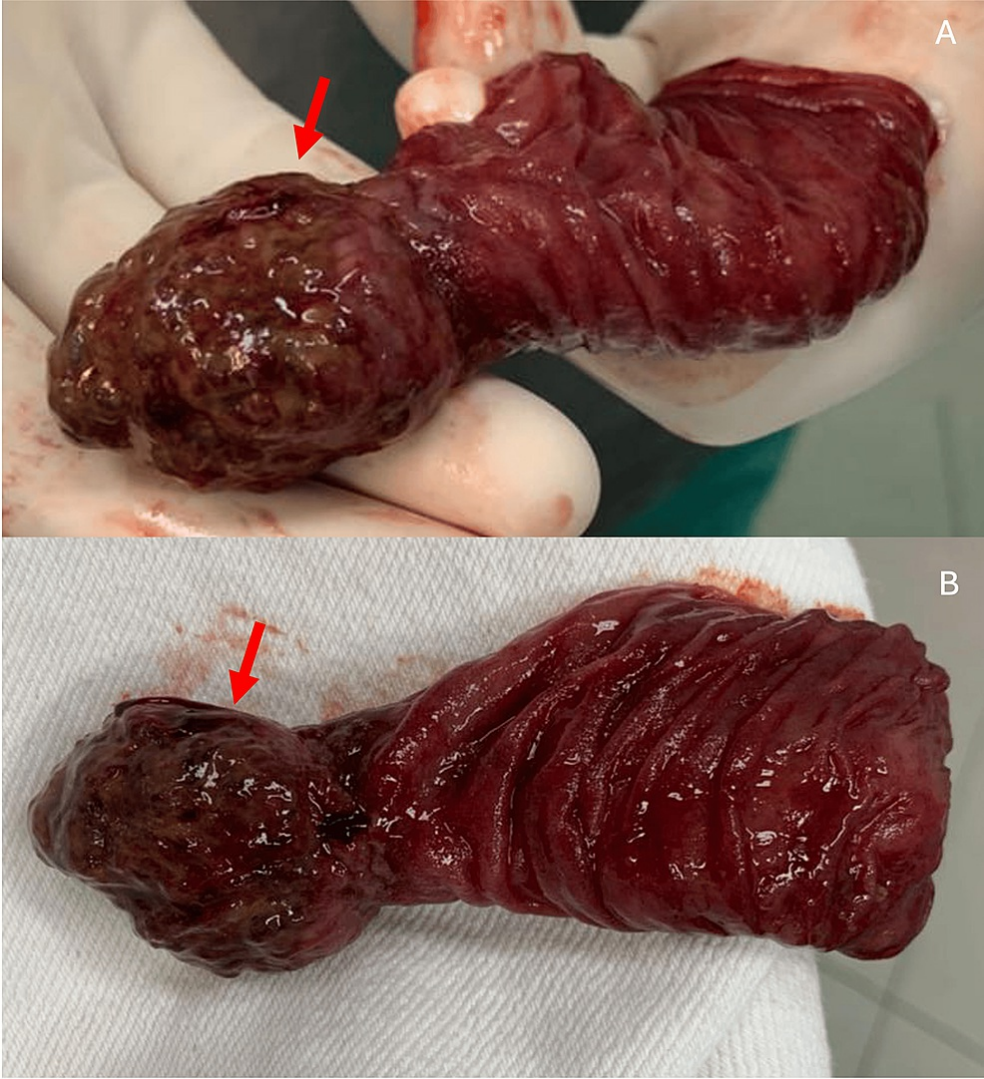
(A and B) This round lesion had a hardened consistency and a brown color and functioned as an intussusception lead point (red arrow)

A manual reduction of intussusception was performed. After the maneuver, the presence of a tumor in the jejunal wall was noted, measuring 3.5 cm in its longest axis. This lesion had a hardened consistency and a brown color and functioned as an intussusception lead point (Figure 3). Segmental enterectomy was performed with manual end-to-end primary anastomosis in two planes with 3-0 Vicryl and 3-0 cotton. A review of the abdominal cavity showed no other changes.

The result of the pathology revealed a fusocellular lesion with a low nuclear grade favoring a gastrointestinal stromal tumor. The surgical margins were free. The immunohistochemical report showed negativity for the DOG-1 and cKIT markers, associated with diffuse expression of desmin and smooth muscle actin (1A4) compatible with intestinal leiomyoma.

The patient evolved satisfactorily in the postoperative period, being discharged on the 10th day after surgery. She was referred for outpatient follow-up and is doing well.

## Discussion

Intestinal invagination, or intussusception, is an uncommon finding in adults, representing only 5% of all intussusceptions and 1% of all intestinal obstructions [2]. In adults, 70% to 90% of cases demonstrated a lead point, which is a well-defined neoplastic change as the etiology in 65% of cases [2]. The exact mechanism by which intussusception occurs is not clear. It is believed that lesions in the intestinal wall cause irritation inside the organ's lumen, which can lead to an alteration in peristaltic activity and start the invagination process [2].

Approximately 90% of cases in adults have a well-defined pathology that functions as a lead point, which can be benign (benign tumors, inflammatory lesions, Meckel diverticulum, appendix, or adhesions) or malignant lesions [2]. In the small intestine, malignant, primary, or metastatic lesions represent 14% to 47% of invagination cases [2]. Symptoms tend to be more chronic or intermittent, such as abdominal pain (71%), nausea and vomiting (68%), abdominal distension with partial obstruction (45%), or a mass palpable on physical examination [3].

CT with oral and intravenous contrast is widely recognized as the most accurate diagnostic tool (58-100% accuracy in recent series) [4]. The alternation of the hyper- and hypodense layers of the intestinal wall gives the telescoped segment the appearance of the classic “target signal” [4].

The treatment of intussusception in adults is always surgical [2]. The current debate revolves around intraoperative management; primary resection versus invagination reduction followed by a more limited resection is discussed [2]. The latter is discouraged in the suspicion of malignancy due to the possibility of intraluminal seeding, venous embolization in the areas of ulcerated mucosa, and complications in the anastomosis [2].

Leiomyomas are the most common symptomatic benign tumors of the small intestine, with a peak incidence in individuals between 50 and 60 years of age [5]. The jejunum is the most frequent location of leiomyomas, followed by the ileum and duodenum [5]. Four different growth patterns are observed: intraluminal, extraluminal, and dumbbell-shaped [5]. Microscopically, leiomyomas consist of well-differentiated smooth muscle bands with no evidence of mitosis [5]. The absence of mitosis is a critical parameter to rule out malignancy (leiomyosarcoma) [5].

Pathologists have recently changed the names leiomyoma and leiomyosarcoma to the term stromal tumor [5]. Typically, immunohistochemistry of gastrointestinal stromal tumors expresses the marker CD117 (KIT), while smooth muscle actin expression (1A4) is more frequent in stromal tumors of the small intestine [6].

Most leiomyomas are asymptomatic [5]. Due to the tendency of these lesions to be highly vascularized and ulcerated, digestive hemorrhage is its most frequent presentation when symptomatic (65%), particularly in the duodenum [5]. Obstructive or intussusception conditions are the second most frequent form of presentation (25%) [5]. Occasionally, these tumors can reach large dimensions and become palpable in asymptomatic patients [5].

The preoperative diagnosis of leiomyomas is difficult, partly due to its rare occurrence and partly due to the absence of pathognomonic symptoms [5]. An increase in the number of diagnoses of leiomyomas is expected due to the greater number of enteroscopies performed [5]. The most important prognostic factors in small bowel tumors are early diagnosis and complete surgical resection [7].

## Conclusions

We emphasize the importance of a detailed clinical evaluation of patients with an abdominal condition who seek emergency care. The cause of intestinal obstruction is a diagnostic challenge due to the numerous pathologies that can lead to the development of the condition. Guided anamnesis, detailed physical examinations, and accurate subsidiary exams that do not delay diagnosis are the cornerstones of emergency room care. Knowing the ideal time to refer the patient to the operating room requires knowledge and practice. The case reported in this article with the final diagnosis of jejunal leiomyoma as a cause of intestinal intussusception is surprising for its rarity and illustrates the range of pathologies that can lead to intestinal obstruction.
